# Recombinant tandem multi-linear neutralizing epitopes of human enterovirus 71 elicited protective immunity in mice

**DOI:** 10.1186/1743-422X-11-79

**Published:** 2014-05-06

**Authors:** Yue-Xiang Li, Hui Zhao, Rui-Yuan Cao, Yong-Qiang Deng, Jian-Feng Han, Shun-Ya Zhu, Jie Ma, Long Liu, E-De Qin, Cheng-Feng Qin

**Affiliations:** 1 Graduate School, Anhui Medical University, Hefei 230032, Anhui, China; 2 Department of Virology, State Key Laboratory of Pathogen and Biosecurity, Beijing Institute of Microbiology and Epidemiology, Beijing, China

**Keywords:** Enterovirus 71, Vaccine, Linear neutralizing epitopes

## Abstract

**Background:**

Human Enterovirus 71 (EV71) has emerged as the leading cause of viral encephalitis in children, especially in the Asia-Pacific regions. EV71 vaccine development is of high priority at present, and neutralization antibodies have been documented to play critical roles during *in vitro* and *in vivo* protection against EV71 infection.

**Results:**

In this study, a novel strategy to produce EV71 vaccine candidate based on recombinant multiple tandem linear neutralizing epitopes (mTLNE) was proposed. The three well identified EV71 linear neutralizing epitopes in capsid proteins, VP1-SP55, VP1-SP70 and VP2-SP28, were sequentially linked by a Gly-Ser linker ((G_4_S)_3_), and expressed in *E.coli* in fusion with the Trx and His tag at either terminal. The recombinant protein mTLNE was soluble and could be purified by standard affinity chromatography. Following three dosage of immunization in adult mice, EV71-specific IgG and neutralization antibodies were readily induced by recombinant mTLNE. IgG subtyping demonstrated that lgG1 antibodies dominated the mTLNE-induced humoral immune response. Especially, cytokine profiling in spleen cells from the mTLNE-immunized mice revealed high production of IL-4 and IL-6. Finally, *in vivo* challenge experiments showed that passive transfer with anti-mTLNE sera conferred full protection against lethal EV71 challenge in neonatal mice.

**Conclusion:**

Our results demonstrated that this rational designed recombinant mTLNE might have the potential to be further developed as an EV71 vaccine in the future.

## Introduction

Human enterovirus 71 (EV71), a typical single-stranded, positive-sense RNA virus, belongs to the *Enterovirus* genus of the *Picornaviridae* family. In recent years, EV71 has emerged as the most important causative agent of Hand, Foot and Mouse disease (HFMD) affecting mostly young children, especially those younger than 5 years old. The clinical symptoms of EV71 infection include simple exanthema, serious aseptic meningitis, acute flaccid paralysis as well as brainstem encephalitis [1]. Although present in most countries, the largest outbreaks of disease have been seen in the Asia-Pacific region over the past 15 years [2-6], and many areas have experienced cyclical epidemics that occur every 2–3 years [7-10]. EV71 infection has now been recognized as an important global public health issue.

Vaccination probably offers the best option for disease control, but there is no available licensed vaccine against EV71. Several vaccine candidates including formaldehyde-inactivated whole-virus vaccine, live-attenuated vaccine, virus-like particles (VLPs), DNA vaccine and subunit vaccine, have showed promise for clinical use [11-20]. Especially, inactivated EV71 vaccines manufactured in mainland China have undergone phase III clinical trials with ideal efficacy [21].

The genome of EV71 is about 7.4 kb in length, which first encodes a long polyprotein with a single open reading frame followed by a poly A tract. Then, the polyprotein is divided into three different precursor proteins (P1, P2 and P3). The P1 precursor protein is further cleaved to four structural proteins, including VP0 (the precursor of VP2 and VP4), VP3 and VP1, which make up the capsid; whereas P2 and P3 are cleaved to non-structural proteins that are involved in genome replication and translation [22]. Among the capsid protein, VP1, VP2, and VP3 are external, and well exposed to host immune system. Neutralizing antibodies against EV71 have been demonstrated as the most important factors in limiting the severity of infection [23]. Several linear neutralizing epitopes have been identified within the capsid proteins of EV71. Foo and colleagues have characterized two neutralizing epitopes in VP1, SP55 (amino acids 163–177) and SP70 (amino acids 208–222), both were capable of eliciting neutralizing antibodies and conferred protection against homologous and heterologous EV71 strains in neonatal BALB/c mice [24]. Moreover, monoclonal antibody targeting the linear epitope on VP1 protein, spanning amino acids 215–219, provided full protection against EV71 challenge *in vivo *[25,26]. Recently, screening with overlapping synthetic peptides covering the entire capsid protein of EV71 identified SP28 (amino acids 136–150 of VP2) as potential neutralizing epitope [27]. Epitope-based vaccines containing well-defined protective epitopes are supposed to stimulate effective and specific protective immune responses while avoiding potential undesirable side-effect [28,29]. Various epitope-based vaccine candidates against virus infection have been well demonstrated with ideal immunogenicity and protection [30-32], and several promising pre-clinical and clinical trials for vaccines that involve peptide-based strategies are currently being carried out [33,34], yet only a few epitope-based EV71 vaccines have been described [24,35,36].

In this study, a novel EV71 epitope-based vaccine candidate was designed through tandem connecting the three known EV71 neutralizing epitopes with the Gly-Ser linker and expressed in *E.coli*. This recombinant multiple tandem linear neutralizing epitopes, named mTLNE, was evidenced to evoke humoral and cellular immune responses and passive transfer with anti-mTLNE sera conferred full protection against lethal EV71 challenge in mice.

## Results

### Expression and characterization of recombinant mTLNE

**Figure 1:**
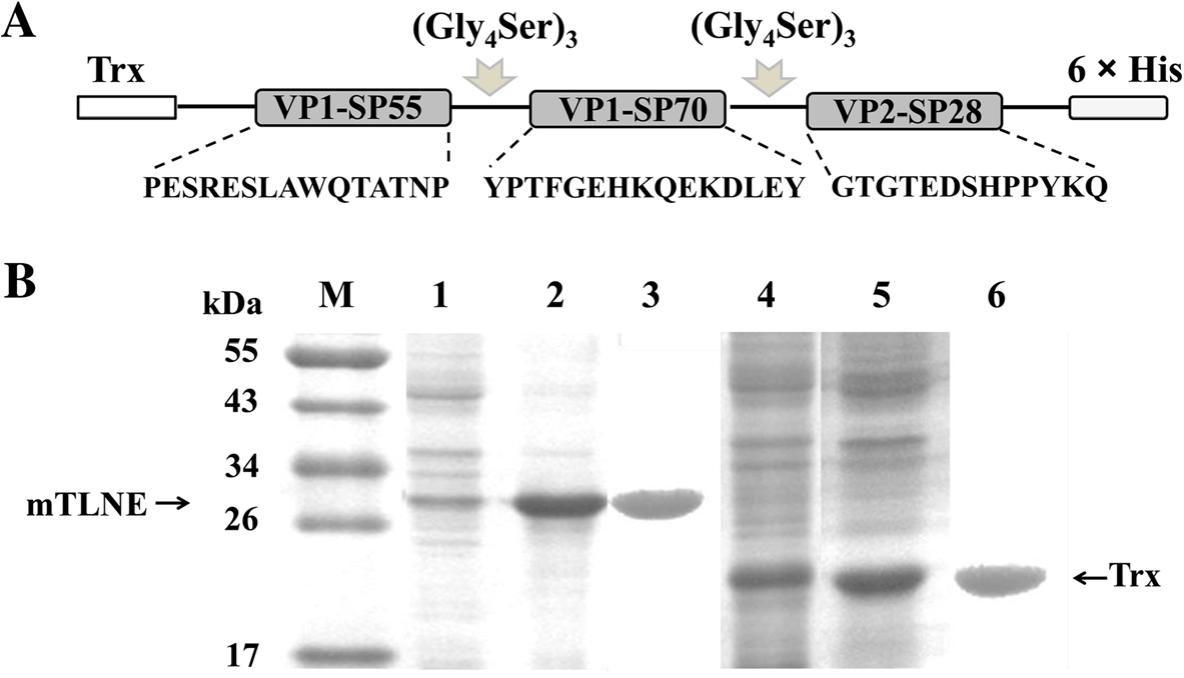
**Expression and purification of recombinant mTLNE. (A)** Schematic representation of the mTLNE constructs. Three linear neutralizing epitopes (VP1-SP55, VP1-SP70 and VP2-SP28) was sequentially linked with (Gly_4_Ser)_3_ sequence. Thioredoxin (Trx) was fused at the N-terminal, and His-Patch at the C-terminal. **(B)** SDS-PAGE results of recombinant proteins. The induced cells were harvested by centrifugation, and the pellet was re-suspended completely by mixing in PBS. Following sonication, the supernatant and the precipitate were harvested and assessed on by SDS-PAGE. Prominent protein bands of about 30 kDa and 20.8 kDa were visible in induced fractions. Lanes 1 and 2: the precipitate and supernatant from *E. coli* receiving pET32a-mTLNE. Lanes 4 and 5: the precipitate and supernatant from *E. coli* receiving pET32a plasmid. Lanes 3 and 6: the purified recombinant mTLNE and Trx protein.

The three known EV71 linear neutralizing epitopes (VP1-SP55, VP1-SP70 and VP2-SP28) were sequentially connected by a linker ((Gly_4_Ser)_3_) and inverted into the expression plasmid pET32a (Figure 1A). Trx was fused at the N-terminal to enhance solubility and immunogenicity, and the C-terminal His-Patch was included for purification on metal-chelating resin. As shown in Figure 1B, the expressed mTLNE was approximately 30 kDa (lane 1 and 2), and the control Trx was about 20.8 kDa (lane 4 and 5) as expected. The majority of recombinant proteins retained in supernatant after sonication (lane 2), indicating that mTLNE was mainly in the form of solubility in *E.coli*. After a single purification with Ni-NTA agarose, recombinant mTLNE was isolated as a single band (lane 3). Thus, soluble mTLNE was highly expressed in *E.coli* and easily purified by standard affinity chromatography.

**Figure 2:**
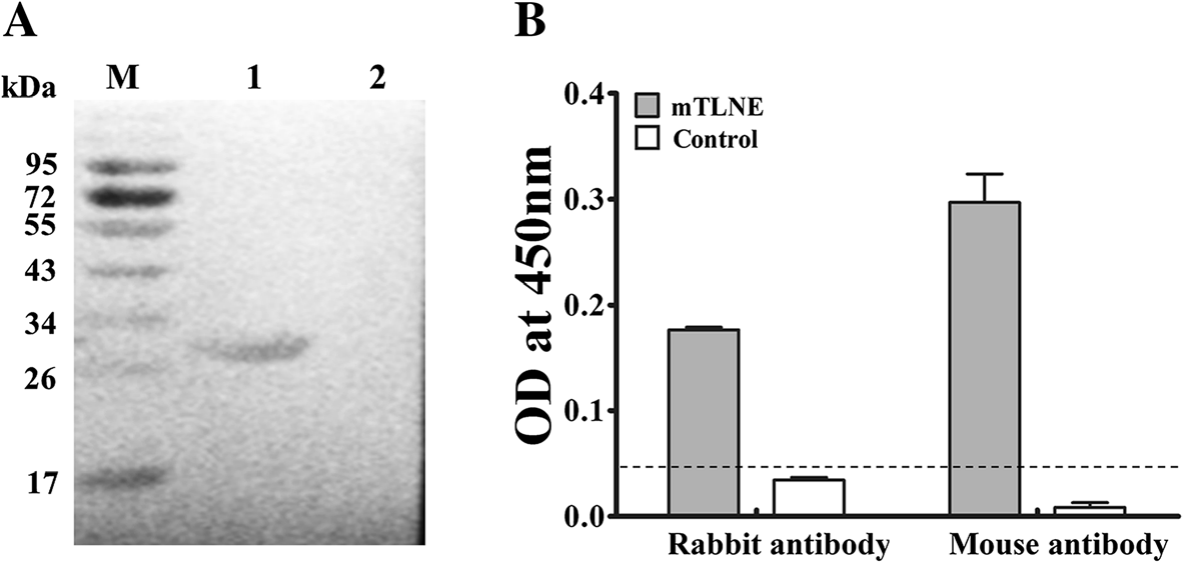
**Serological characterization of recombinant mTLNE. (A)** Western blotting assay using mouse polyclonal antibody against EV71. Lanes 1: mTLNE proteins; Lanes 2: Trx proteins. **(B)** ELISA using rabbit and mouse polyclonal antibodies against EV71. Recombinant mTLNE could be specifically recognized by the corresponding mouse and rabbit antibodies compared with the control Trx. The cut-off value for the ELISA is shown by a dotted line.

Western blotting assay was performed to characterize the antigenicity of mTLNE. The result showed that mTLNE could be recognized by mouse anti-EV71 polyclonal antibody (Figure 2A). Further analysis with ELISA confirmed that recombinant mTLNE could specifically react with mouse and rabbit antisera against EV71 (Figure 2B), meanwhile the control protein Trx had only an insignificant background reading. These data suggested that recombinant protein mTLNE possessed good immune reactivity, and was potential to be an EV71 antigen as originally designed.

### Recombinant mTLNE induced EV71-specific humoral and cellular immune response in mice

**Figure 3:**
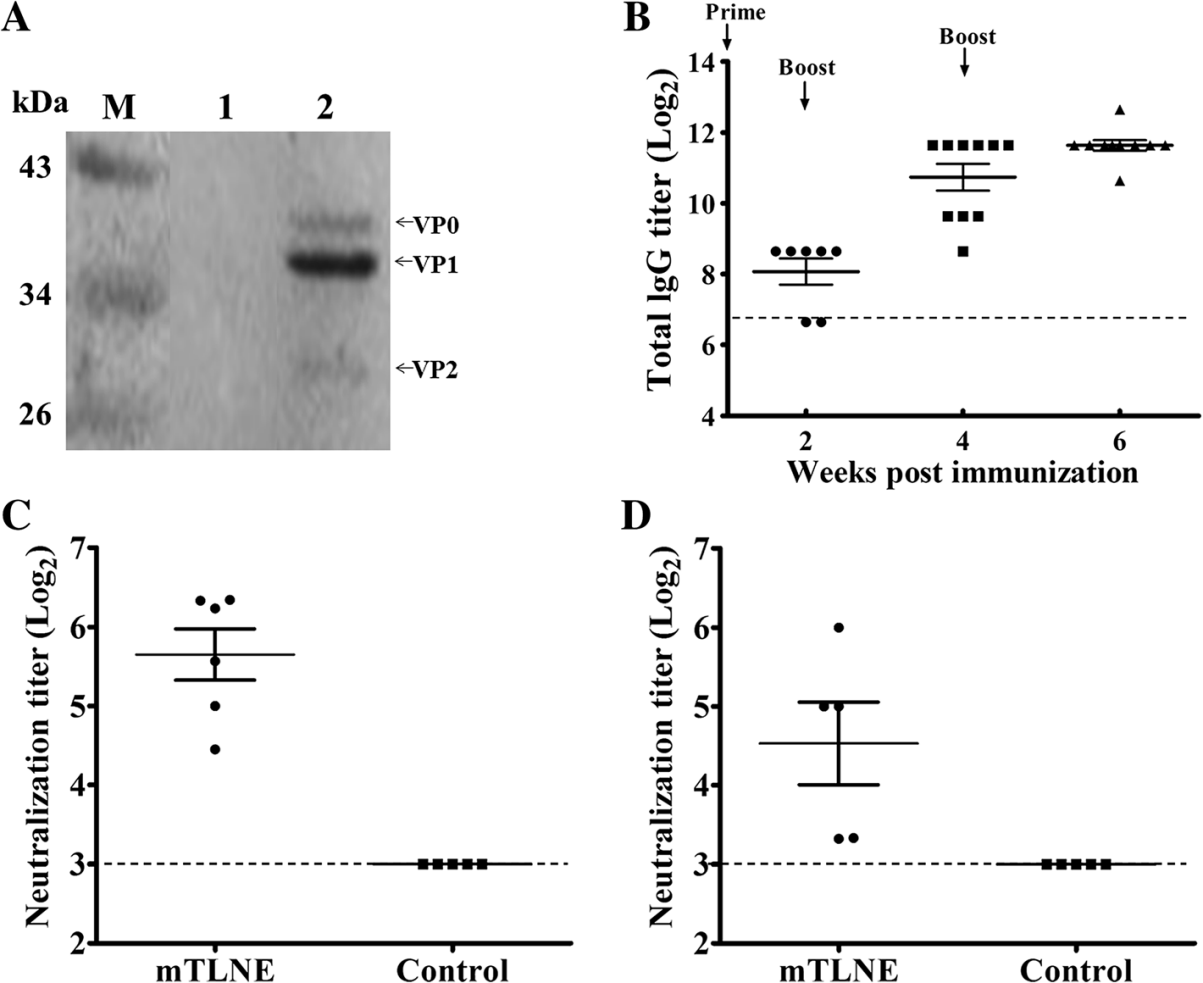
**Humoral immune response in mice immunized with mTLNE. (A)** The immunoreactivity of serum from mice immunized with mTLNE was determined by Western blotting. The total proteins of EV71 were harvested from infected cell lysate, and the cell lysate from the uninfected RD cells was used as control. Lanes 1: uninfected RD cell lysate. Lanes 2: the total proteins of EV71. The position of the three capsid proteins of EV71 (VP0, VP1 and VP2) was indicated by arrow, respectively. **(B)** The lgG antibody titer against EV71 was measured by IFA at two weeks after each immunization. **(C and D)** Neutralization antibody titer against EV71 strain AH08/06 (genotype C4) and prototype strain BrCr (genotype A) were measured by microneutralization assay at two weeks after the final immunization. Dotted lines represent limits of detection.

To test the immunogenicity of recombinant mTLNE in mice, groups of BALB/c mice were immunized with mTLNE three times at two-week intervals. Group of mice immunized with equal dose of Trx protein was set as control. Serum samples were collected at two weeks after each immunization and EV71-specific antibody response were determined accordingly. Firstly, the immuno-reactivity of the anti-mTLNE sera was determined by Western blotting using EV71 virions (Figure 3A). The results showed that the anti-mTLNE sera were reactive with three EV71 capsid proteins, including VP0 (35 kDa), VP1 (32 kDa) and VP2 (28 kDa). The results confirmed that immunization with mTLNE was capable of inducing antisera recognizing VP1 and VP2 proteins of EV71.

**Figure 4:**
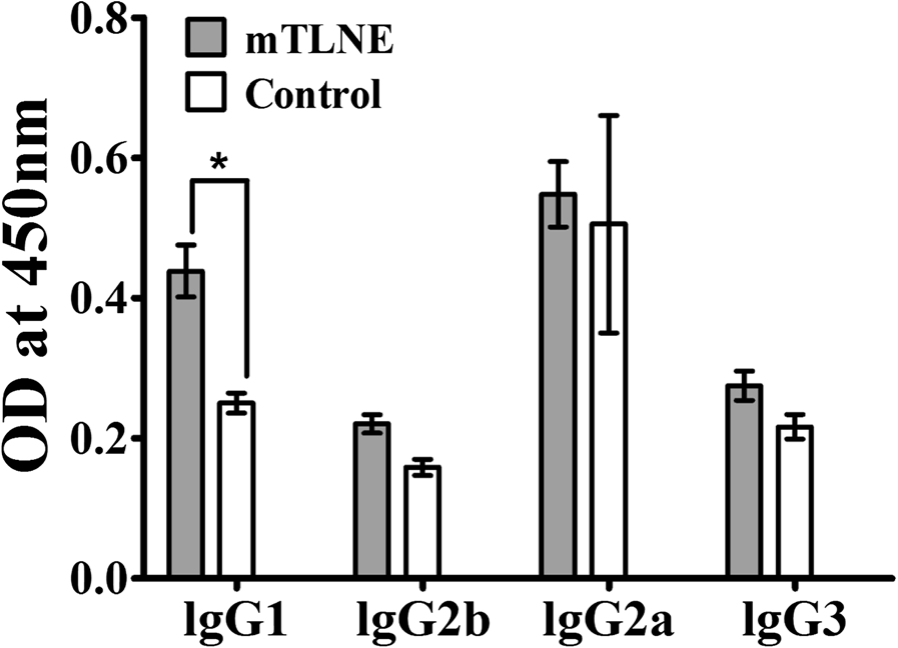
**IgG subtype induced by mTLNE.** Profile of IgG subtype in immune sera from mice immunized with mTLNE. Serum samples were collected at two weeks after the last immunization and analyzed for presence of IgG1, IgG2a, IgG2b and IgG3 subtype antibodies by ELISA. (*) IgG1 subtype antibodies of sera from mTLNE group were statistically significant compared with control group using *t*-test.

Then, EV71-specific IgG antibody response was measured by indirect immunofluorescence assay (IFA). The results showed that IgG antibodies were readily induced in each mTLNE-immunized mice at two weeks post prime immunization, and the IgG titers gradually increased along with boost immunizations. After the third immunization, the lgG titer peaked at 1:3191, and no detectable IgG antibody was observed in mice immunized with Trx as expected (Figure 3B). Subsequently, ELISA was performed to characterize the antibody reactivity to each individual EV71 epitope. The results revealed that higher titers of IgG antibody against the three epitopes (VP1-SP55, VP1-SP70 and VP2-SP28) were induced in the mTLNE-immunized mice (Table 1). IgG subtyping results showed that the level of IgG1 in mTLNE-immunized mice was significantly higher than that of control group, while no significant difference was observed for IgG2a, IgG2b and IgG3 (Figure 4). Furthermore, neutralizing antibodies assay showed that three doses of mTLNE immunization elicited high titer neutralizing antibodies against both genotype C4 (Figure 3C) and A strains of EV71 (Figure 3D), while no EV71-specific neutralizing antibodies were detected in all mice immunized with Trx.

**Table 1:** ELISA titers against individual EV71 epitope

	**Synthetic peptides**		
	**VP1-SP55**	**VP1-SP70**	**VP2-SP28**
mTLNE	1:246	1:1488	1:1710
Control	<1:100	<1:100	<1:100

Epitope specific IgG antibody titers of sera in two weeks after the last boost were assayed by ELISA, using corresponding unconjugated synthetic peptide as the capture antigen. Data represents the geometric mean titer of individual serum sample (n = 10) harvested from the immunized mice.

**Figure 5:**
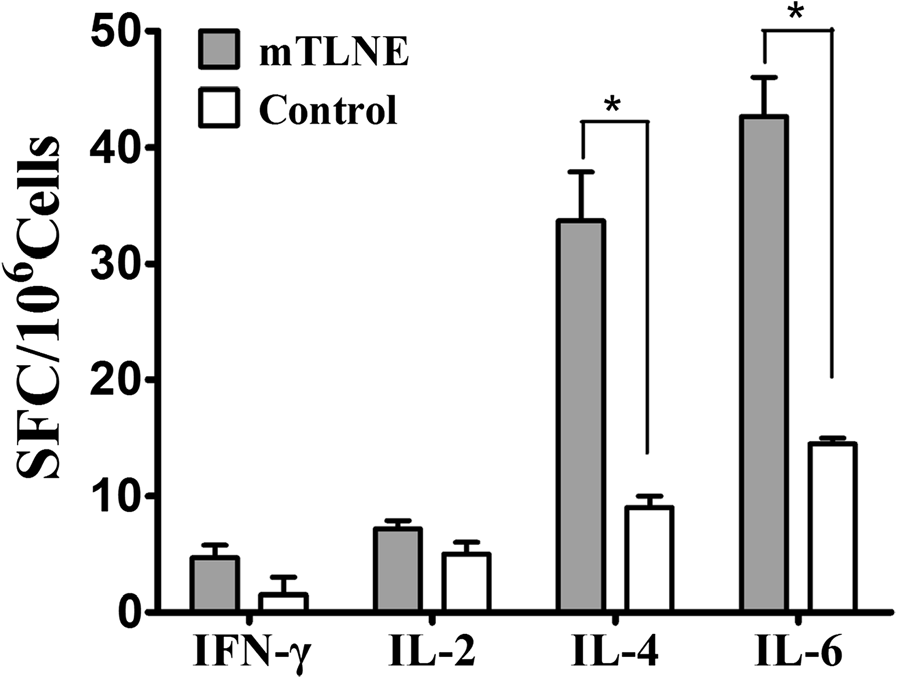
**Cytokine profile in mice immunized with mTLNE.** Cytokines response of splenocytes from mice immunized with mTLNE was measured by ELISPOT. The numbers of spot-forming cells (SFC) from mTLNE-immunized mice were compared with that from the control group. (*) SFC secreting cytokines (IL-4 and IL-6) from mTLNE group were statistically significant compared with control group using *t*-test.

To make sure whether cellular immunity was induced by mTLNE, splenocyte proliferative responses in mice immunized with mTLNE were determined by measuring the levels of IFN-γ, IL-2, IL-4 and IL-6. ELISPOT assay showed that high level of IL-4 and IL-6 were predominantly produced in mTLNE-immunized mice in comparison to the control group. Meanwhile, no significant difference was observed in IL-2 and IFN-γ production between mTLNE-immunized and Trx-immunized mice (Figure 5). Taken together, these results demonstrated immunization with recombinant mTLNE were capabale of inducing EV71-specific humoral and cellular immune response in mice.

### Passive transfer with anti-mTLNE confers full protection against lethal EV71 challenge in neonatal mice

**Figure 6:**
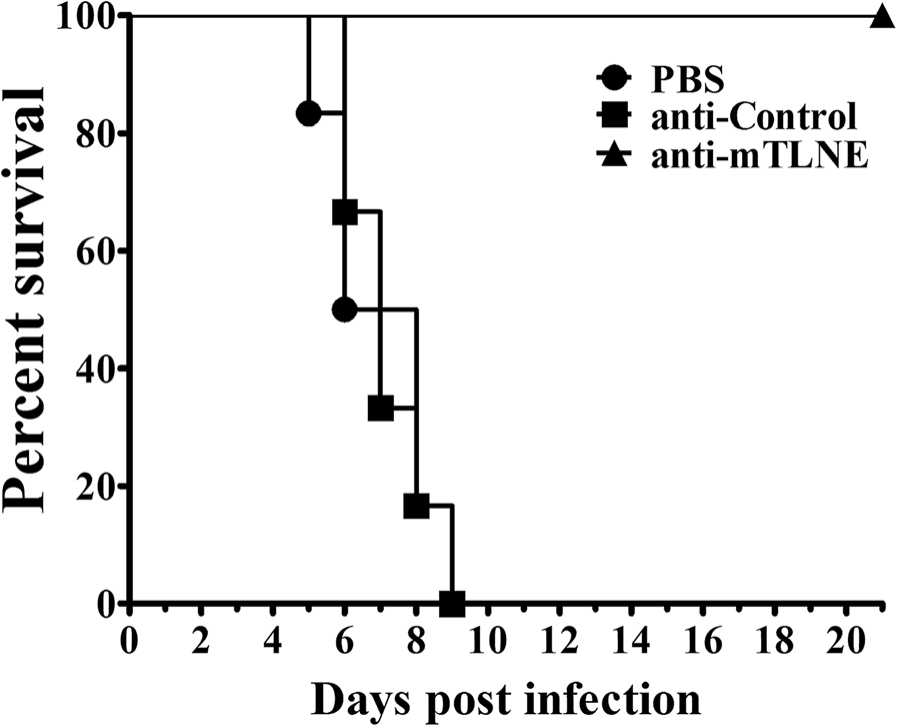
**Passive protection conferred by anti-mTLNE sera.** Groups of one-day-old BALB/c mice were inoculated i.c. with 10 LD_50_ of EV71, followed by i.p. administration of anti-mTLNE sera at 4 h post inoculation. PBS and anti-Trx sera were set as controls. The mortality was monitored for 21 days. Log-rank test were performed to analyze the statistical significant.

Finally, the *in vivo* protective efficacy against lethal EV71 challenge was evaluated in an established neonatal mice model [37]. Mice were i.c. inoculated with lethal dose of EV71 and then i.p. administered with anti-mTLNE sera. Mice treated with PBS and anti-Trx sera were set as controls. As shown in Figure 6, all the mice treated with anti-mTLNE sera survived after the challenge, and none of them developed any clinical manifestations; meanwhile all mice that received anti-Trx sera or PBS became hairless lesions and paralysis of limbs at 5 days post-infection and all died within 10 days. Log-rank analysis showed that anti-mTLNE sera significantly prolonged the survival time and prevented EV71-caused mortality in mice in comparison with the controls. Together, these results demonstrated passive transfer with anti-mTLNE sera provided full protection against EV71 challenge in neonatal mice.

## Discussion

Epitope-based vaccine provides an opportunity to rationally engineer specific epitopes to increase potency and breadth and avoid the potential side-effect of other unrelated epitopes that contribute few to protection [38,39]. To data, only a few epitope-based vaccines against EV71 have been described. In our study, we propose a novel strategy to connect the three identified linear neutralizing peptides by a Gly-Ser linker, generating the recombinant epitope-based vaccine candidate mTLNE. Recombinant mTLNE was efficiently expressed in *E. coli* system and easily purified with affinity chromatography. The Gly-Ser linker was chosen to increase the flexibility of the recombinant protein, to minimize interference between adjacent epitopes, and to facilitate forming natural conformation [40]. The designed mTLNE contained two epitopes in VP1 and one epitope in VP2, and antisera from mice immunized with mTLNE interacted simultaneously with both VP1 and VP2 proteins of EV71 (Figure 3A).

Further, immunization with mTLNE induced potent humoral immune responses as demonstrated by the elicitation of EV71-specific IgG antibodies (Figure 3B). Antibodies induced by mTLNE immunization were able to neutralize both the circulating strain AH08/06 and the prototype strain BrCr (Figure 3D), suggested that mTLNE might induce broad neutralization against homologous and heterologous genotypes of EV71. This is consistent with previous reports by Li et al. [41]. The specific IgG antibody response in mice appeared somewhat divergent among different epitopes, with relative low titer against VP1-SP55 (Table 1). Such immunogenic inferiority of SP55 epitope has also been observed by others [42]. These differences are likely a reflection of the nature of viral antigen.

In general, neutralizing antibodies are important index for protection against EV71 infection. In the present study, the geometric mean of neutralizing antibody titers induced by mTLNE was 50, which was higher than that of single synthetic peptide-SP70 [24]. Compared with inactivated vaccines, the neutralizing titers elicited by mTLNE were relatively low due to lack of other antigens [17,37]. However, the advantage of this epitope based vaccine approach should not be highlighted. Especially, antibody-dependent enhancement (ADE) of EV71 infection has been observed in both experimental and clinical setting [41,43-45]. Recent findings from human intravenous immunoglobulin clarified the distinct function of each IgG subtype on neutralization and enhancement of EV71 infection [46], and IgG1 subtype is deemed to dominate neutralization while IgG3 associates with ADE. The robustness of IgG1 response, not IgG3, induced by mTLNE (Figure 4) indicated potential benefits for protection. Most importantly, our data clearly demonstrated that passive transfer of anti-mTLNE sera conferred full protection to neonatal mice against lethal EV71 challenge (Figure 6).

In addition to humoral immunity, mTLNE immunization induced cellular immune responses as evidenced by elevated production of specific cytokines (Figure 5). The production of IL-4 and IL-6 in mTLNE-immunized mouse splenocytes suggested a Th2 immune response. In mice, IL-4 and IL-6 generally switches activated B cells to the IgG1 subtype, which was confirmed by IgG subtypes assay and IgG1 subtype was prevalent in case of mTLNE group (Figure 4). These observations suggest that mTLNE contain mainly B-cell epitopes, which has also been observed by Foo et al. [24,36]. Currently, only a few T-cell epitopes on the capsid proteins of EV71 have been identified [47,48]. In the future work, a peptide-based vaccine containing multiple B-cell epitopes as well as T-cell epitopes can be expected, which might be an ideal vaccine that are capability of inducing a protective antibody response and a cytotoxic T-cell response important for killing infected host cells.

Overall, we propose a novel epitope-based vaccine approach by connecting linear neutralizing peptides, and primary immunogenicity and protection experiments in mice demonstrated the potential for further development as EV71 vaccine.

## Materials and methods

### Cell lines and viruses

Human Rhabdomyosarcoma RD cells and African green monkey kidney Vero cells were cultured in DMEM medium (Invitrogen) containing 10% fetal bovine serum (FBS) (Hyclone) at 37°C in the presence of 5% CO_2 _[46,49]. EV71 prototype strain BrCr (GenBank accession no.U22521), belongs to genotype A, and genotype C4 strain AH08/06 (GenBank accession no. HQ611148.1) was isolated from an HFMD patient during an outbreak in 2008 in Anhui, China [50]. The EV71 virus stocks were propagated in RD cells according standard protocol and titers were determined in Vero cells [35,51].

### Preparation of mouse and rabbit polyclonal antibody against EV71

The mouse polyclonal antibody against EV71 was prepared in our lab [41] and the rabbit polyclonal antibody against EV71 were prepared as followed. A rabbit (2.5-3 kg) was immunized subcutaneouly (s.c.) with heat-inactivated EV71 (2 × 10^7^ PFU/ml) employing Freund’s complete adjuvant (Sigma), and boosted with the same dose antigen with Freund’s incomplete adjuvant (Sigma). Two weeks after the boost immunization, serum samples were titrated and stored frozen until use.

### Overlap PCR amplification and plasmid construction

The cDNA fragments encoding three tandem EV71 neutralizing epitopes (VP1-SP55, VP1-SP70 and VP2-SP28) which connected by the Gly-Ser linker ((Gly_4_Ser)_3_) were amplified by overlap PCR. The primers and reaction conditions are available upon request. The PCR products were purified and inserted into the pET32a vector (Novagene) to generate the recombinant plasmid pET32a-mTLNE. The recombinant plasmid was confirmed by restriction enzyme digestion and DNA sequencing.

### Protein expression and purification

The plasmid pET32a and pET32a-mTLNE was transformed in BL21 Chemically Competent cells (TIANGEN). The transformed BL21 cells were incubated with shaking at 37°C until OD_600_ reaches 0.6, then induced with 0.1 mM IPTG (Sigma) at 37°C for 6 h. The cells were harvested by centrifugation, and the pellet was re-suspended completely by mixing in phosphate-buffered saline (PBS). Following ultrasonication, recombinant mTLNE was purified through a Ni-NTA agarose (KWBIO, China) according to manufacturer’s instructions, and the concentration of was determined by a bicinchoninic acid Protein Assay Kit (Pierce).

### SDS-PAGE and Western blotting assay

All samples were mixed with loading buffer and loaded onto a homogeneous 12% polyacrylamide gel. Western blotting analysis was performed using the corresponding antibodies described as above. In brief, the PVDF membranes were incubated with the corresponding antibody at room temperature for 1 h, followed by blocking in 10% skim milk overnight at 4°C. A horse-radish-peroxidase (HRP)-conjugated secondary antibody at a dilution of 1:5000 was used to detect the primary antibody. The membranes were subjected to three washes for 10 min each time in PBS containing 0.05% Tween 20 and developed with ECL Western substrate (Bio-Rad).

### Enzyme-linked immunosorbent assay (ELISA)

The 96-well microtiter plates were coated with coating buffer (pH 9.6) containing 1 μg/ml of mTLNE overnight at 4°C followed by 10% skim milk blocking. The coated plate was then incubated with rabbit (1:400) or mouse (1:500) anti-EV71 antibody at 37°C for 1 h, and followed by incubating HRP-conjugated secondary antibodies at 37°C for 30 min. A total of 100 μl of TMB peroxidase substrate was added for incubation for 15 min at room temperature. Finally, the absorbance at 450 nm was recorded using an ELISA plate reader.

### Mice immunization

All animal experimental procedures were carried out in strict accordance with and approved by the Animal Experiment Committee of Beijing Institute of Microbiology and Epidemiology. Groups of 6-week-old female BALB/c mice (n = 10) were inoculated with 50 μg of mTLNE protein, or Trx protein (control group) by intraperitoneal (i.p.) injection with Freund’s complete adjuvant (Sigma). All mice were then boosted twice with the same dose in Freund’s incomplete adjuvant (Sigma) at a 2-week interval. Two weeks after each immunization, serum samples were prepared and stored frozen until use.

### Antibody response assay

The titers of IgG antibody in mice sera were detected by IFA as previously described [52]. In brief, confluent RD cells infected with EV71 were harvested and suspended in DMEM containing 10% FBS. The suspended cells were then inoculated onto slides and fixed with acetone for 30 min in -20°C. All serum samples were serially diluted (two fold) in PBS, and mice polyclonal antibodies (1:100 dilution) against EV71 were used as positive control. After incubation for 1 h at 37°C, the slides were washed three times in PBS. Then, Alexa Fluor®488 conjugated goat anti-mouse IgG (Invitrogen) in 0.02% Evans blue was added and incubated for 30 min at 37°C. Finally, positive cells were detected using a fluorescent microscope (Olympus). The IgG subtype profiles in mouse sera were determined with a commercially available mouse immunoglobulin isotyping kit (Invitrogen) according to the manufacturer’s instruction.

The IgG titers of the immunized serum to each epitope (VP1-SP55, VP1-SP70 and VP2-SP28) were determined by ELISA. The unconjugated synthetic peptides representing the amino acid sequence of VP1-SP55, VP1-SP70 or VP2-SP28 were synthesized by Shanghai Bootech Bioscience&Technology Co., Ltd (Shanghai, China). The 96-well microtiter plates were coated with 100 μl of coating buffer containing 15 μg/ml of unconjugated synthetic peptides. All sera samples serially diluted (two fold) in PBS were incubated in triplicate wells (100 μl/well) for 1 h at 37°C. Following procedures were made as described in Methods “ELISA”. IgG antibody titers were expressed by the highest serum dilution at which the absorbance at 450 nm is higher than cut off value.

The neutralizing antibody titers against different genotype of EV71 were measured by microneutralization assay in Vero cells as previously described [53]. The neutralizing antibody titer was calculated using the Reed-Muench method [51].

### Cytokine analysis

The production of cytokine from stimulated splenocytes was detected by enzyme-linked immunospot (ELISPOT) analysis using BD^TM^ ELISPOT Set. In brief, the 96-well plates was coated respectively with IFN-γ, IL-2, IL-4, IL-6 capture antibody overnight at 4°C. Spleen cells (10^6^/well) from the immunized mice were added and cultured at 37°C for 36 h, with total proteins of heat-inactivated EV71 (100 PFU/well) or ConA (250 ng/well, Sigma). Following procedures were made according to the manufacturer’s instruction.

### *In vivo* challenge experiments

The neonatal mice model of EV71 infection has been described [37]. Groups of BALB/c neonatal mice (n ≥ 5) were intracranially (i.c.) inoculated with 10 LD_50_ of EV71 strain AH08/06, and 40 μl of anti-mTLNE or anti-Trx serum was i.p. administered at 4 h post infection. PBS was set as negative control. The mortality was then monitored for at least 21 days.

### Statistical analysis

The survival curve of each group was compared by using the Log-rank test with GraphPad Prism 5.0, and the other data were analyzed using *t*-test, and *p* < 0.05 was considerate as significance.

## Competing interests

The authors declare that they have no competing interests.

## Authors’ contributions

Conceived and designed the experiments: YXL HZ CFQ. Performed the experiments: YXL HZ SYZ. Analyzed the data: YXL HZ RYC YQD JFH CFQ. Contributed reagents/materials/analysis tools: JM LL SYZ EDQ. Wrote the paper: YXL HZ CFQ. All authors read and approved the final manuscript.
